# Morphological and Histological Analysis of the Gastrointestinal Systems in *Triplophysa strauchii* and *Triplophysa tenuis*: Insights into Digestive Adaptations

**DOI:** 10.3390/ani15081095

**Published:** 2025-04-10

**Authors:** Zhengwei Wang, Lirong Zhang, Jie Wei, Huimin Hao, Syeda Maira Hamid, Shixin Gao, Wenjun Li, Zhulan Nie

**Affiliations:** 1 College of Life Science and Technology, Tarim University, Alar 843300, China; 10757232145@stumail.taru.edu.cn (Z.W.); 18379996700@163.com (L.Z.); 120070006@taru.edu.cn (J.W.); 10757223077@stumail.taru.edu.cn (H.H.); 1107572024402@stumail.taru.edu.cn (S.M.H.); follow_spot@163.com (S.G.); 18080583487@163.com (W.L.); 2 Xinjiang Production & Construction Corps Key Laboratory of Protection and Utilization of Biological Resources in Tarim Basin, Alar 843300, China

**Keywords:** *Triplophysa strauchii*, *Triplophysa tenuis*, digestive system, morphology, histology, feeding habit

## Abstract

Fish are vital for material cycling and energy flow in aquatic ecosystems. The genus *Triplophysa*, with over 100 known species, is significant in the Central Asian highlands’ freshwater ecosystems. *T. strauchii* and *T. tenuis*, as representatives, occupy distinct ecological niches and face challenges from climate change and human activities. There is a lack of research on *Triplophysa* fishes’ digestive systems, especially comparative studies, so this research aims to fill this gap. In September 2024, 40 samples of *T. strauchii* were collected from Sayram Lake and 40 samples of *T. tenuis* were collected from the Muzat River in Xinjiang. After acclimation, morphological observations (measuring fish and digestive tract parameters) and histological analyses (paraffin sectioning, HE staining, and microscopy) were carried out. The data were sorted in Excel and analyzed with an independent samples *t*-test in SPSS 27.0. Morphologically, *T. strauchii* has an obtuse snout, terminal mouth, specific upper lip papillae, and an S-shaped intestine about (1.45 ± 0.11) times its body length, while *T. tenuis* has an arc-shaped subterminal mouth, fringed papillae, and a spiral-shaped intestine around (0.82 ± 0.09) times its body length. Both possess a digestive tract, glands, and a hepatopancreas attached to the mesentery. Histologically, a large number of club cells were found in the oropharyngeal cavities of both species; their secretions have an adhesive effect on food, aiding food selection. Their digestive systems vary in structure and cell composition: the oropharyngeal cavity has three layers; the esophagus has four layers with more goblet cells in *T. strauchii*; the stomach has three regions without goblet cells and a thicker muscular layer in *T. strauchii*; the intestinal wall has four layers with different villi and goblet cell distributions; the hepatopancreas has lobules; and *T. strauchii* has a typical portal area. In conclusion, this study systematically compared the gastrointestinal systems of *T. strauchii* and *T. tenuis* for the first time, revealing significant structural differences related to their niches and feeding patterns as adaptations to specific environments. It fills the research gap, provides a basis for exploring fish ecological adaptation and environmental impacts on digestion, offers new ideas for *Triplophysa* protection strategies, and guides fish evolutionary biology research and *Triplophysa* resource protection and utilization.

## Simple Summary

Fish are vital for aquatic ecosystems. The *Triplophysa* genus, with over 100 species, is key to the Central Asian highlands’ freshwater ecosystems. Research on the digestive systems of *T. strauchii* and *T. tenuis*, facing threats in different niches, is scarce. In 2024, samples were collected and analyzed. It was found that their digestive systems vary, adapting to niches, which filled knowledge gaps.

## 1. Introduction

Fish play an important role in the material cycling and energy flow within aquatic ecosystems. The genus *Triplophysa*, as an important group of the Cobitidae family in the Cypriniformes order, contains more than 100 known species and holds a significant position in the freshwater ecosystems of the Central Asian highlands [1]. As highlighted in [2], these fishes hold a pivotal position in the freshwater ecosystems of the Central Asian highlands. Their distribution spans the Qinghai–Tibet Plateau and its geologically complex periphery. Their habitats include rivers, lakes, streams, and high-altitude cold springs [3], with marked variations in altitude, water temperature, water quality, and flow velocity driving substantial niche differentiation among *Triplophysa* species [4].

*Triplophysa strauchii* and *T. tenuis*, representative *Triplophysa* species, occupy distinct ecological niches in complex ecosystems [5]. Historically, *T. strauchii* thrived in Tianshan’s high-altitude lakes with gravel/sandy bottoms and high dissolved oxygen [6]. However, climate-change-induced water-level fluctuations in recent decades have shrunk suitable spawning and foraging areas, leading to a slight population decline in some regions. To cope, *T. strauchii* evolved a flattened body, which aids stability in water flow, flexible foraging in rock crevices, and predator avoidance [7]. Conversely, *T. tenuis* was originally widespread in low-altitude lake shallows and slow-flowing streams. Yet, human activities like agricultural water diversion and pollution have fragmented its habitats. *T. tenuis* is active in plant-rich lake shallows and stream downstream areas, with diverse food sources [8]. To adapt to changing habitats and food availability, it developed a slender body for easy movement through aquatic plants [9]. The overexploitation of small aquatic animals has increased the proportion of plant-based food in its diet, prompting an increase in cellulase and amylase activity for better plant-debris digestion. Moreover, *T. strauchii* has expanded its habitat to new shallower and warmer water bodies at lake edges. Its visual system has evolved to be more sensitive to low light in these new habitats. *T. tenuis*, meanwhile, has developed a stronger antioxidant defense system to survive in polluted waters. It has also become more tolerant of water-temperature fluctuations, enabling survival in areas with wide-ranging temperatures throughout the year.

The digestive system, as the core organ system for fish to obtain nutrients and maintain life activities, is of great significance for in-depth research [10]. The structure and function of the digestive system are directly related to the efficiency of fish in ingesting, digesting, and absorbing food, and thus affect their growth and development, reproductive capacity, and competitiveness in the ecosystem [11]. Through morphological and histological studies of the fish digestive system, we can not only gain an in-depth understanding of their feeding habits and nutritional physiological mechanisms but also provide important clues for exploring the ecological adaptability and evolutionary history of fish [12].

Currently, research on the digestive systems of *Triplophysa* fish is relatively scarce, especially comparative studies among different species. Moreover, previous research has mainly been confined to investigations of individual species, which only enable one to form a one-sided understanding of the digestive systems of *Triplophysa* fish. For example, although some studies have described the basic anatomical features of individual species, they have failed to establish clearly the connection between the characteristics of the digestive system and ecological factors. Additionally, there is an insufficient understanding of the correspondence between the evolutionary process of the digestive systems of *Triplophysa* species and their long-term adaptation to different environmental conditions [7,8]. This study aims to fill this knowledge gap by comparing the digestive systems of *T. strauchii* and *T. tenuis*.

In this study, we collected samples of *T. strauchii* and *T. tenuis* from their natural habitats, and then, in terms of morphological analysis, measured and compared their body lengths, body weights, as well as the key morphological parameters of their digestive tracts. Histological research involved preparing tissue sections of their digestive organs, staining them with appropriate dyes, and observing the cellular structures under an optical microscope. *T. strauchii*, adapted to high-altitude cold-water environments with limited food resources, may have evolved an optimized digestive system structure for efficient nutrient absorption, while *T. tenuis*, living in habitats with more diverse food sources, is likely to possess a more adaptable digestive system. We expected to find differences between these two species in aspects such as the length and complexity of their digestive tracts and the structure of their digestive glands, which are highly likely to be closely related to their unique ecological requirements. Therefore, this study aims to conduct a detailed morphological and histological comparison of the digestive systems of *T. strauchii* and *T. tenuis*, fill the research gap in this field, and provide a solid theoretical basis for biological research, resource protection, and the rational development and utilization of fish species in the genus *Triplophysa*.

## 2. Materials and Methods

### 2.1. Experimental Materials

In September 2024, researchers utilized cage traps and gillnets with a mesh size of 2a = 2 cm to conduct a sample collection at Sayram Lake (44°39′36″ N, 81°15′36″ E) and Muzat River (41°28′12″ N, 80°59′00″ E) in Xinjiang, successfully gathering 40 specimens of *T. strauchii* from the former and 40 specimens of *T. tenuis* from the latter. Sayram Lake, encircled by Tianshan mountain ranges like the Korgurqin Mountains, has a relatively straight, trapezoidal shoreline and marked bottom-depth variations. Its water, boasting a transparency exceeding 10 m, ranking among the highest in transparency among lakes in China [13], provides a suitable habitat where *T. strauchii* typically inhabit the gravel crevices. The Muzat River originates from Mount Khan Tengri on the southern slopes of the Tianshan Mountains. It is the largest tributary of the Weigan River and is mainly replenished by glacial meltwater from high altitudes [14]. The river is rich in gravel, sand, and aquatic animals and plants, which provides an ideal habitat for *T. tenuis*. The collection strictly followed the guidelines of Reference [15] to ensure fish integrity and health while minimizing stress responses. After collection, the live specimens were swiftly transported to the laboratory and placed in aquariums outfitted with advanced oxygenation and water-temperature control systems, which accurately replicate the fishes’ natural habitats by simulating natural environmental parameters.

During the 2–3 day acclimation period, dissolved oxygen was 6–8 mg/L and water temperature was 16–25 °C, with a 12 h light/12 h dark cycle (500–1000 lux). We provided natural food sourced from fish habitats. We collected natural plankton and benthos from the water bodies of these habitats following standard sampling protocols. After collection, these organisms were brought back to the laboratory and temporarily cultured in a 15 L water body with the same environmental conditions as those for the fish during the acclimation period. Moreover, approximately 60% of the samples collected were plankton, while benthos accounted for about 40%. To maintain the quality of natural food, we monitored water quality parameters daily, such as dissolved oxygen, pH value, and ammonia nitrogen content. Any signs of deterioration or contamination were carefully monitored and dealt with immediately. The feeding frequency was three times a day. Before each feeding, we thoroughly stirred the water body in which the natural food was temporarily cultured to ensure the uniform distribution of plankton and benthos. Each time, we fed the fish with 1.5–1.8 L of this water body. Behaviors and body conditions were monitored by cameras and software, while a water quality monitor was used to adjust the rearing conditions. After measurement and analysis, *T. strauchii* had a body length of 98.46 ± 19.20 mm and weight of 13.26 ± 6.47 g; *T. tenuis* had a body length of 93.89 ± 11.35 mm and weight of 12.72 ± 5.69 g. Finally, 15 healthy and active fish from each species were selected for subsequent experiments.

### 2.2. Morphological Observation

Before the experiment, the experimental fish were anesthetized with 35 mg/L MS-222 (Fujian Jinjiang Aquatic Products Co., Ltd., Jinjiang, China) to ensure they were in a pain-free and quiet state [16]. After anesthesia, the body weight of the fish was accurately measured using an electronic balance with an accuracy of 0.01 g. Key morphological indicators, such as the total length and body length, were carefully measured using a vernier caliper with an accuracy of 0.01 mm, and then these data were recorded. During dissection, precise tools and techniques were employed to carefully open the abdominal cavity, and the digestive system, including the digestive tract (oropharynx, esophagus, stomach, and intestine) and the hepatopancreas, was completely removed. The length of each segment of the digestive tract was measured using a vernier caliper and a ruler, and the ratio of the digestive tract length to the body length (intestine–length ratio) was precisely calculated to quantify the morphological differences in the digestive systems of the two species of *Triplophysa*.

### 2.3. Histological Analysis

Tissue blocks of about (5 × 5 × 3) mm were excised from each segment of the digestive tract and from the liver and pancreas and immediately fixed in Bouin’s fixative (Beijing Bio-laboratech Co., Ltd., Beijing, China) for 24 h. After fixation, according to the conventional histological procedure, we dehydrated the tissue blocks through a graded alcohol series (Tianjin Zhiyuan Chemical Reagent Co., Ltd., Tianjin, China). They were successively immersed in 70%, 80%, 90%, 95%, and 100% alcohol solutions for 1–2 h each. Then, we cleared the dehydrated tissue blocks with xylene (Shanghai Macklin Biochemical Co., Ltd., Shanghai, China) for 30 min to 1 h to prevent the tissue from becoming brittle. After that, we embedded the tissue blocks in molten paraffin, ensuring proper positioning and alignment. We used a YD-202 microtome to cut sections with a thickness of 5–7 μm. The sections were stained with hematoxylin and eosin (HE) and observed under a microscope. We used image analysis software (Motic Images Plus 2.0) to measure histological parameters such as the height of mucosal folds and the thickness of the muscular layer in each segment of the digestive tract. On each section, we selected five random fields of view for each parameter and calculated the average value to ensure the accuracy and reliability of the data [17].

### 2.4. Data Processing

We expressed the data obtained from the experiment as “mean ± standard deviation (SD)”. First, we used Excel software to group, organize, and classify the data, preparing them for further statistical analysis. Then, we carried out an independent samples *t*-test using SPSS 27.0 statistical analysis software to test whether there were significant differences between different groups of data. If the independent samples *t*-test results showed significant differences between groups, we calculated effect size measures like Cohen’s d to better understand the magnitude of the differences. Throughout the statistical analysis, we set the significance level at 0.05. That is, when *p* < 0.05, we considered the differences between groups to be statistically significant.

## 3. Results

### 3.1. Morphology of the Digestive System

**Figure 1:**
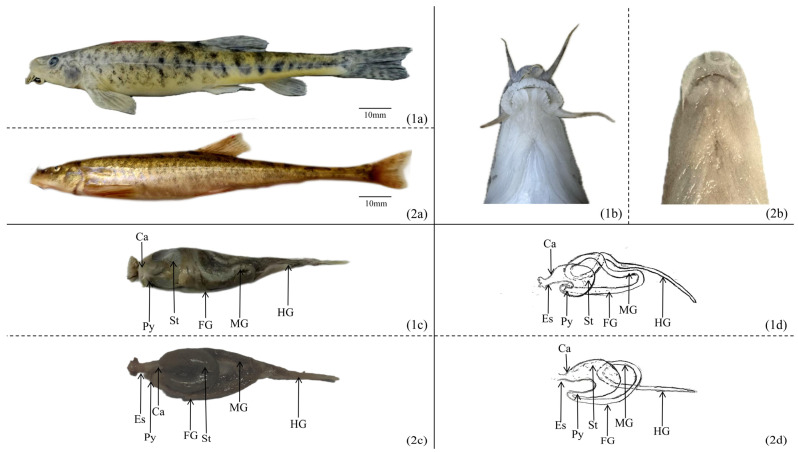
The morphology of the digestive system in *T. strauchii* and *T. tenuis*: (**1a**) morphology of *T. strauchii*; (**2a**) morphology of *T. tenuis*; (**1b**) mouth morphology of *T. strauchii*; (**2b**) mouth morphology of *T. tenuis*; (**1c**) digestive tract of *T. strauchii*; (**2c**) digestive tract of *T. tenuis*; (**1d**) diagram of the intestinal pattern of *T. strauchii*; (**2d**) diagram of the intestinal pattern of *T. tenuis*. Es: esophagus; Ca: cardia; St: stomach; Py: pylorus; FG: foregut; MG: midgut; HG: hindgut.

The digestive systems of *T. strauchii* and *T. tenuis* are both composed of the digestive tract and digestive glands. The digestive tract includes the oropharyngeal cavity (since there is no clear boundary between the oral and pharyngeal cavities, they are collectively named), esophagus, stomach, and intestine (divided into the anterior, middle, and posterior intestines). *T. strauchii* has an obtuse snout and a terminal oral cavity. Its upper lip is densely studded with papillae: one row at the front edge and two or three rows at the corners. The lower lip is thick and full of deep folds. There is a deep median groove reaching the isthmus, with a single papilla on each side. The spoon-shaped lower jaw does not protrude beyond the lips. It also has three pairs of barbels. The rostral barbels can reach the mouth corners, the maxillary barbels can extend to the anterior or inferior margins of the eyes, and the mandibular barbels can reach or surpass the posterior margins of the eyes (Figure 1(1a,1b)). *T. tenuis* has an arc-shaped inferior oral cavity; its upper lip features one or two rows of fimbriated papillae (Figure 1(2a,2b)). The esophagus of both species, which connect the oropharyngeal cavity and the stomach, are relatively short. Their U-shaped stomachs consist of the cardiac part, body, and pyloric part. The intestine of *T. strauchii* first bends forward behind the stomach. Then it twists into an S-shape at the pelvic fin base before reaching the anus. Its intestinal length is approximately (1.45 ± 0.11) times its body length (Figure 1(1c,1d)). By contrast, the intestine of *T. tenuis* is spirally shaped like the Greek letter “*φ*”. It can be divided into the anterior, middle, and posterior intestines. The part from the pylorus to the first turn is the anterior intestine, the second turn is the middle intestine, and the rest is the posterior intestine (Figure 1(2c,2d)). It gradually tapers from front to back, with an intestinal length of about (0.82 ± 0.09) times the body length. In both species, the pancreas fuses closely with the liver to form the hepatopancreas, which adheres tightly to the mesentery and covers the ventral surface of the digestive tract.

### 3.2. Histology of the Digestive System

#### 3.2.1. Oropharyngeal Cavity

**Figure 2:**
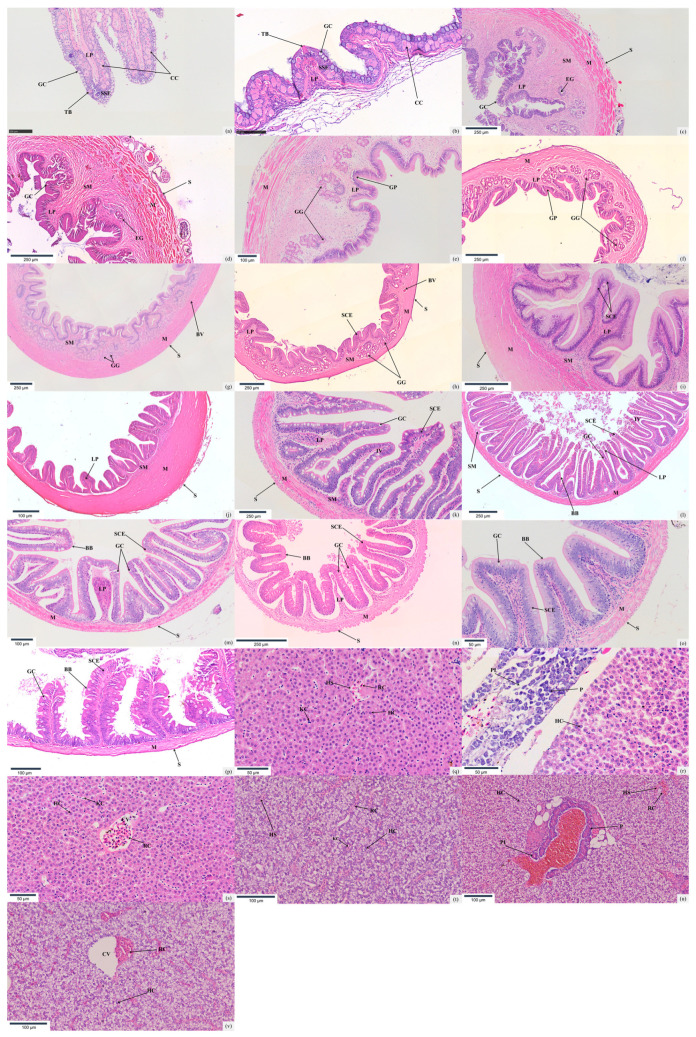
The histology of digestive systems in *T. strauchii* and *T. tenuis*: (**a**,**c**,**e**,**g**,**i**,**k**,**m**,**o**) stand for the oropharyngeal cavity, esophagus, cardia, stomach, pylorus, foregut, midgut, and hindgut of *T. strauchii*, respectively; (**b**,**d**,**f**,**h**,**j**,**l**,**n**,**p**) stand for the oropharyngeal cavity, esophagus, cardia, stomach, pylorus, foregut, midgut, and hindgut of *T. tenuis*, respectively. (**q**–**s**) The hepatopancreas of *T. strauchii*; (**t**–**v**) the hepatopancreas of *T. tenuis*. TB: taste bud; M: muscular; LP: lamina propria; GC: goblet cell; SSE: stratified squamous epithelium; CC: club cell; SM: submucosa; EG: esophageal gland; S: serosa; GP: gastric pit; GG: gastric gland; SCE: single columnar epithelium; HS: hepatic sinusoid; KC: hepatic macrophages; RC: red blood cells; P: pancreas; PI: pancreatic islets; CV: central vein; BB: brush border; IV: intestinal villus; BV: blood vessels.

The oropharyngeal cavities of *T. strauchii* and *T. tenuis* are situated at the anterior terminus of the digestive tract. Structurally, both feature a tri-layer architecture, namely the mucosal layer, submucosal layer, and muscular layer. The mucosal layer, composed of stratified squamous epithelium, contains a profusion of club cells. These cells are distributed interstitially among epidermal cells, and their volume is conspicuously larger than that of other cells. Additionally, scattered bottle-shaped taste buds populate the mucosal layer. These taste buds penetrate the epithelial layer; their apical ends open into the oropharyngeal lumen, while their basal portions are supported by protrusions of the lamina propria. The submucosal layer consists of loose connective tissue. Meanwhile, the muscular layer is constructed of circular striated muscles. Within this muscular layer, adipose tissue, blood vessels, and connective fibers are randomly dispersed. (Figure 2a,b).

#### 3.2.2. Esophagus

The digestive tract walls of *T. strauchii* and *T. tenuis*, starting from the esophagus, are successively divided into four layers from the outer surface to the inner side, namely the serosa, muscularis, submucosa, and mucosa. The serosa is composed of mesothelial cells and thin connective tissue. The muscularis consists of circumferential striated muscle fibers with adipose tissue, blood vessels, and connective tissues interspersed within it. The submucosa, made up of loose connective tissue, contains longitudinal skeletal muscle fibers extending into the lamina propria. In the mucosa layer, the number of goblet cells in *T. strauchii* is greater than that in *T. tenuis* (Table 1). The mucosa projects into the esophageal lumen as longitudinal, non-branching folds that resemble intestinal villi (Figure 2c,d).

#### 3.2.3. Stomach

The stomachs of *T. strauchii* and *T. tenuis* can be distinctly demarcated into the cardiac region, the corpus ventriculi, and the pyloric segment. Their mucosal epithelium consists of densely arranged single-layer columnar cells, with most cell nuclei positioned at the basal part of the cells. Throughout the entire course of observation, no goblet cells were detected. From the cardiac region to the pyloric segment, the fold height of the stomachs in these two *Triplophysa* species manifests a consistently upward-climbing trend, and the fold width initially escalates gradually and then tapers off. Many of their gastric glands assume a long-oval configuration, and some are even stretched into slender, tube-like structures. The muscular layer is relatively well-developed and is composed of the inner circular smooth muscle and the outer longitudinal smooth muscle, both of which belong to the smooth-muscle category (Figure 2e–j). The thickness of the muscular layer in *T. strauchii* is notably greater than that in *T. tenuis* (*p* < 0.05) (Table 1).

#### 3.2.4. Intestine

The intestinal wall structures of *T. strauchii* and *T. tenuis* are both composed of the mucosa layer, submucosa layer, muscular layer, and serosa layer. In the foregut region, the mucosa layer of both fish species protrudes significantly into the intestinal lumen, forming many slender and closely arranged intestinal villi. *T. strauchii* exhibits an intestinal villi count of 18.40 ± 2.07 per square millimeter, while *T. tenuis* possesses a larger number of intestinal villi, with a count of 24.60 ± 2.17 per square millimeter. Shifting the focus from the foregut to the midgut, branched secondary mucosal folds can be observed. *T. strauchii* has an intestinal villi count of 18.90 ± 3.07 per square millimeter in its midgut, and *T. tenuis* shows an intestinal villi count of 20.10 ± 3.14 per square millimeter in the same area. As for the hindgut, the intestinal villi of *T. strauchii* are curved, roughly triangular, and accompanied by many secondary mucosal folds, with 12.70 ± 1.25 per square millimeter. In contrast, the intestinal villi in the hindgut of *T. tenuis* are more spread out, with a larger distance between adjacent villi, and the number is 15.00 ± 1.76 per square millimeter. In addition, the number of goblet cells in the hindgut of *T. strauchii* is significantly higher than that of *T. tenuis* (*p* < 0.05) (Table 1). The intestinal mucosal epithelium of both species of highland loaches is mainly composed of single-layer columnar absorptive cells, among which vacuolated goblet cells are randomly scattered. The number of goblet cells increases from the foregut to the hindgut. As the intestine extends from the anterior to the posterior part, the height of the mucosal folds in the mucosa layer gradually diminishes. Meanwhile, the nuclei of the epithelial cells remain steadily at the base of the cells, and a striated border is spontaneously formed at the free end. The columnar cells gradually become shorter in height, and their quantity decreases correspondingly. *T. strauchii* has a significantly larger number of goblet cells in its midgut compared to *T. tenuis*, but the situation is reversed in the hindgut. From the anterior to the middle and posterior parts of the intestine, both the height and width of the intestinal villi of the two loach species decrease remarkably. The thickness of the submucosa layer increases gradually. The thickness of the muscular layer of *T. strauchii* first increases and then decreases from the front to the back, while that of *T. tenuis* shows a continuous decline (Figure 2k–p).

#### 3.2.5. Hepatopancreas

The hepatopancreas of *T. strauchii* and *T. tenuis* consists of hepatic lobules composed of round hepatocytes. Owing to the under-developed connective tissue between the hepatic lobules, their structure is not distinct. Inside the hepatic lobules, there are central veins. Moreover, these central veins are radially encircled by cord-like hepatic plates formed by polygonal hepatocytes, with hepatic sinusoids interspersed among them. The central veins are irregular in shape and randomly distributed, and their walls feature openings connecting to the hepatic sinusoids. The nuclei of hepatocytes are round, situated in the middle, and the nucleoli are prominent. The endothelial cells of the hepatic sinusoids are flat and thin, and within the lumen, a small number of hepatic macrophages and blood cells can be found. In the portal area of the liver of *T. strauchii*, one can observe interlobular arteries with thick walls, interlobular bile ducts whose walls are made up of low-columnar epithelial cells, and interlobular veins characterized by thin walls and irregular lumens. In contrast, the portal area of the liver in *T. tenuis* is atypical; it shows less-defined organization of the typical vascular and ductal structures compared to *T. strauchii*. On the liver surface of both species, a small amount of pancreatic tissue can be detected. In the exocrine part, the acinar cells are predominantly short-columnar, possessing large and round nuclei. Inside the acinar lumen, small Centroacinar cells with lightly stained cytoplasm are visible. The endocrine part is represented by the islets of Langerhans, which are clusters of cells separated from the exocrine part by connective tissue (Figure 2q–v).

## 4. Discussion

### 4.1. The Correlation Between the Morphology of the Digestive System and Feeding Habits

The significant morphological differences in the digestive systems of *T. strauchii* and *T. tenuis* are closely linked to their feeding habits [18,19]. For *T. strauchii*, its blunt rounded snout, terminal mouth, upper lip covered with papillae, thick wrinkled lower lip, and three pairs of barbels are typical features for benthic feeding. In high-altitude lakes with complex bottoms [20,21], the sensory nerve endings of the papillae and the barbels help detect prey such as small invertebrates and organic detritus. They can quickly locate hidden small crustaceans and capture them using the special structures of their lips, making the feeding habit of *T. strauchii* biased towards omnivory with benthic feeding as the main mode, while also showing a certain carnivorous tendency. Research [22] indicates that this morphology is the result of the long-term adaptation to benthic life and is crucial for obtaining food in its ecological niche. *T. tenuis* has an arc-shaped subterminal oral cavity and one or two rows of fimbriated papillae on its upper lip. The arc-shaped oral cavity facilitates its search for prey near limited shelters or substrate surfaces. The fimbriated papillae, like fine tweezers, can precisely capture small zooplankton, aquatic insect larvae, etc. [23]. This set of oral structural characteristics indicates that *T. tenuis* has highly adapted to preying on small aquatic animals during the process of evolution. Some studies also show [20] that this kind of oral structure is common in some fish species that mainly feed on small aquatic animals and is an important evolutionary feature for adapting to preying on small prey. In comparison to select congeners within the genus *Triplophysa*, such as *T. brevviuda*, this species exhibits a conspicuously wide mouth. This morphological trait equips it to effectively scrape algae and organic matter adherent to rocks. The observed feeding morphology strongly indicates an herbivorous feeding preference [24]. Evidently, this represents a distinct deviation from the feeding strategies adopted by *T. strauchii* and *T. tenuis*.

The intestinal morphology of *T. strauchii* and *T. tenuis* vividly reflects their distinct feeding habits. *T. strauchii*, dwelling in high-altitude lakes with scarce and unstable food resources, mainly preys on small invertebrates [25], yet it has a relatively long S-shaped intestine, about 1.45 times its body length, unlike typical carnivorous fish with shorter intestines and pyloric caeca. In fact, it is an omnivorous species with a carnivorous bias, consuming small invertebrates as well as complex organic detritus with indigestible parts. The long intestine provides sufficient digestion time and space, enhancing the utilization of limited food and enabling the exploitation of diverse food sources [26]. The intestinal villi and microvilli increase the absorptive surface area [27], fitting its diet. Moreover, studies [28,29] confirm that a relatively long intestine can boost the nutrient utilization rate of omnivorous fish on complex diets, thus helping *T. strauchii* survive and reproduce in its ecological niche despite lacking pyloric caeca.

*Triplophysa tenuis* has a short, spiral-shaped intestine, approximately 0.82 times its body length, and it lacks pyloric caeca. This is consistent with its simple and easily digestible carnivorous diet mainly consisting of small zooplankton and soft-bodied invertebrates [30,31], and while it also reflects its omnivorous characteristics, it has a stronger carnivorous bias. The short length of the intestine matches the fast digestion of its food, and the spiral shape increases the surface area of the intestine. Even without pyloric caeca, this unique intestinal structure helps it efficiently extract nutrients from high-quality animal-based foods to meet the needs of frequent feeding and growth. Research [32] shows that the spiral-shaped intestine is common in carnivorous fish that feed on small and easily digestible prey, optimizing digestion and absorption in their ecological niche. This also means that for *T. tenuis*, the spiral intestine compensates for the absence of pyloric caeca in fulfilling its digestive functions, further illustrating its omnivorous diet with a carnivorous bias. In contrast, the intestinal length of *T. rosa* is 0.38 times its body length, and that of *T. wulongensis* is 0.43 times its body length [33]. The intestines of *T. rosa* and *T. wulongensis* are relatively shorter, and their feeding habits are carnivorous.

### 4.2. Histological Characteristics and Digestive Functions

In the oropharyngeal cavity structure, the mucosal epithelium of *T. strauchii* and *T. tenuis* is composed of stratified squamous epithelium and contains a large number of club cells. This structure forms a key functional unit of the oropharyngeal cavity. The club cells are significantly larger in volume than other cells and are scattered among the epidermal cells [33]. Their secretions have a certain adhesive effect on food and can help select food. Similarly, there are also numerous club cells in the oropharyngeal cavity of *Botia superciliaris* [34]. Mucosal epithelium also has keratinized cells, protecting against mechanical damage during foraging [35]. Abundant mucous cells secrete mucus for food lubrication and initial digestion [36]. Taste buds, another key feature, allow fish to distinguish food sources, optimizing diet, which is crucial for survival in variable environments [37]. The histological similarity in their oropharyngeal cavities, despite habitat and diet differences, shows the basic needs of the digestion start.

Both *T. strauchii* and *T. tenuis* have esophageal structures (serosa, muscular layer, submucosa, and mucosa) that are well adapted for food transport from the oropharyngeal cavity to the stomach [19]. The striated muscles in the muscular layer, under voluntary control, precisely regulate swallowing, ensure smooth food passage, and prevent choking or regurgitation. Neural-induced muscle contractions propel food down the esophagus [38]. Longitudinal skeletal muscle fibers in the submucosa work with circular muscles in the muscular layer to generate peristalsis, facilitating food movement and mixing it with mucosal mucus to reduce friction [39]. Mucosa-based goblet cells secrete mucus, protecting and lubricating the esophageal lining, reducing friction, and safeguarding the epithelium [40]. *T. strauchii* has more goblet cells. Research [41] shows that animals facing irritating or indigestible food in the long term evolve more goblet cells for protection. For *T. strauchii*, such food may harm the esophageal mucosa, so they need more mucus to maintain esophageal function. Conversely, *T. tenuis*, which feeds primarily on aquatic insects, has fewer goblet cells. This suggests that its diet, which is centered around aquatic insects, has a less irritating effect on the esophageal mucosa [42]. In contrast, *T. bleekeri* typically consumes foods such as rough diatoms. These foods may be more abrasive or difficult to digest, and as a result, *T. bleekeri* has a relatively large number of goblet cells to protect its esophageal mucosa from the potential irritation caused by such foods. Research [25] indicates that *Lates calcarifer*’s goblet cell number decreases in a mild food environment. *T. tenuis* might have evolved other adaptation mechanisms. Given its preference for aquatic insects, its digestive system likely has evolved too. For instance, *Oncorhynchus mykiss*, as [43] shows, has a special digestive enzyme system to efficiently decompose food, lessening esophageal irritation. *T. tenuis* may also have a specific intestinal microbiota to aid digestion, thereby reducing the protection pressure on the esophageal mucosa. Their short esophagus, typical of fish, aligns with most digestion and absorption occurring in the stomach and intestines [39].

The muscular layer of the stomach of *T. strauchii* is well-developed and significantly thicker than that of *T. tenuis* (*p* < 0.05), directly related to its diet [44]. *T. strauchii* consumes small invertebrates with hard shells, needing stronger muscle contractions for food processing. The thick muscular layer enables the stomach to generate powerful grinding forces, breaking down prey into smaller particles for better mixing with gastric juice. This mechanical breakdown, crucial for digestion, increases the food surface area for enzymatic action [39]. In both species, the height and width changes of gastric folds from the cardiac to the pyloric region are closely related to digestion. The increasing fold height towards the pylorus offers a larger surface area for food–enzyme contact, promoting more efficient digestion and nutrient absorption [19]. The absence of goblet cells in the gastric mucosa of both species is a common fish feature, fitting the acidic stomach environment. The stomach, mainly through gastric enzymes like pepsin (functioning optimally in an acidic pH), is responsible for protein digestion [45].

The structures like intestinal villi, mucosal epithelial cells, mucosal folds, submucosa, and muscular layers in different intestinal segments of *T. strauchii* and *T. tenuis* show diverse changes related to digestive functions. In the foregut, *T. tenuis* has more villi and stronger absorption. Research [46] indicates that more villi boosts absorption for digestion, and *T. tenuis* follows this. In the midgut, its villi number advantage aids nutrient uptake, consistent with [47]. In the hindgut, *T. strauchii*’s curved villi with secondary folds help contact residues and reabsorb nutrients, while *T. tenuis’* dispersed villi facilitate residue pushing [48]. For the intestinal mucosa epithelium, goblet cell numbers increase from the fore- to the hindgut. *T. strauchii* has more in the midgut for protection and fewer in the hindgut than *T. tenuis*, related to hindgut function differences. Research [48,49] shows that goblet cell mucus protects mucosa and promotes food movement. From front to back, mucosal fold height and columnar cell size/number changes suit different intestinal segment functions. The submucosa thickens for support. *T. strauchii* is an omnivorous fish with a carnivorous bias. It needs to deal with complex foods such as small invertebrates with hard shells in its daily life, so the thickness of its muscular layer first increases and then decreases. The muscular layer of the foregut thickens to powerfully crush the food, and the muscular layer of the hindgut becomes thinner because the demand for processing residues is relatively low. In this way, it can reasonably allocate energy to adapt to the living environment. In contrast, *T. tenuis* feeds on easily digestible foods such as small zooplankton and aquatic insect larvae, and the thickness of its muscular layer gradually decreases. This change effectively avoids energy waste and is an evolutionary strategy to adapt to simple food sources and improve survival efficiency. Research [28,50] shows that intestinal structure changes match functional division and are related to feeding habits, fully reflected in these two loaches.

In the digestive systems of *T. strauchii* and *T. tenuis*, the hepatopancreas plays a crucial role [26]. The liver is an independent organ with unique physiological functions and is an important digestive organ. The bile secreted by the liver is stored in the gallbladder, and the bile duct opens at the front part of the foregut. After being concentrated in the gallbladder, the bile is secreted into the foregut. Bile can reduce the surface tension of fat and promote the formation of chylomicrons. This significantly increases the contact area between fat and lipolytic enzymes, thus accelerating the digestion of fat. Similar findings have been obtained in studies on *T. yarkandensis* [51], indicating that there may be similarities in the digestive processes of these fish species. In hepatic lobules, central veins, hepatic plates, and sinusoids enable material transport, supporting nutrient uptake and waste excretion and ensuring digestion substance processing. Hepatocyte features show their active role in making digestion-related substances like bile precursors [52]. Sinusoid structure aids exchange, and macrophages maintain internal stability for smooth digestion [53]. In *T. strauchii*, the liver portal area has vessels and ducts that have a clear division of labor, suiting its complex diet and high metabolism. In contrast, *T. tenuis* has a less-typical portal area, which fits its simple diet and metabolism, reflecting long-term adaptive evolution [54,55,56]. The pancreatic tissue on the liver surface has exocrine and endocrine parts. Exocrine acinar cells secrete key digestive enzymes, with Centroacinar cells regulating secretion. The endocrine islets of Langerhans maintain blood glucose, providing stable energy for digestion [57].

## 5. Conclusions

This study conducted morphological and histological analyses of the gastrointestinal systems of *T. strauchii* and *T. tenuis*, revealing significant structural differences between the two species. Morphologically, the oral region, barbels, and intestinal morphology of *T. strauchii* are adaptations to benthic complex food sources. In contrast, the unique oral and intestinal characteristics of *T. tenuis* are beneficial for preying on small aquatic animals. Histologically, differences in the cell composition and structure of various digestive parts further enhance their adaptability to specific foods and living environments. These differences are precise responses to their respective ecological niches and feeding patterns during the long-term evolutionary process. This study not only systematically compares the characteristics of the gastrointestinal systems of fish in the genus *Triplophysa* for the first time, filling the research gap in this field, but also provides an important basis for further in-depth exploration of the molecular mechanisms of fish ecological adaptation, the impact of environmental changes on their digestive systems, and other related studies, contributing to the development of fish evolutionary biology.

## Figures and Tables

**Table 1:** Histological parameters of the digestive tracts of *T. strauchii* and *T. tenuis* (mean ± SD).

Morphological Index	Species	Esophagus	Cardia	Stomach	Pylorus	Foregut	Midgut	Hindgut
Spiral valve height/μm	*T. strauchii*	301.11 ± 96.06	250.65 ± 58.04	260.45 ± 40.02	639.51 ± 62.10 ^a^	408.87 ± 31.87	311.74 ± 55.86 ^a^	141.26 ± 50.04 ^a^
	*T. tenuis*	291.59 ± 30.16	275.07 ± 29.35	277.54 ± 27.50	278.13 ± 57.40 ^b^	387.47 ± 36.69	248.30 ± 30.90 ^b^	218.24 ± 56.61 ^b^
Mucosal fold width/μm	*T. strauchii*	115.66 ± 12.80	116.25 ± 26.08 ^a^	201.92 ± 20.95 ^a^	88.59 ± 9.90 ^a^	75.95 ± 26.94 ^a^	64.08 ± 7.04	92.71 ± 23.66
	*T. tenuis*	119.54 ± 33.33	108.37 ± 20.20 ^b^	150.47 ± 29.51 ^b^	136.60 ± 17.98 ^b^	97.31 ± 19.63 ^b^	87.70 ± 11.41	102.13 ± 9.82
Sub mucosa thick/μm	*T. strauchii*	261.66 ± 71.30	189.69 ± 46.23	205.10 ± 88.27	11.53 ± 4.10	9.64 ± 1.77	12.76 ± 1.63	16.45 ± 4.94
	*T. tenuis*	251.66 ± 80.42	182.69 ± 49.90	193.10 ± 82.72	10.43 ± 3.03	9.34 ± 1.95	11.26 ± 1.71	14.95 ± 4.17
Muscle layer thickness/μm	*T. strauchii*	170.09 ± 13.90 ^a^	88.98 ± 8.91 ^a^	252.18 ± 75.34 ^a^	49.53 ± 4.63 ^a^	49.17 ± 12.09	56.89 ± 11.82 ^a^	25.96 ± 2.60
	*T. tenuis*	106.22 ± 6.49 ^b^	64.57 ± 11.90 ^b^	74.42 ± 12.22 ^b^	104.64 ± 7.68 ^b^	51.96 ± 7.55	39.02 ± 5.88 ^b^	28.39 ± 4.09
Goblet cell (cell number/100 μm)	*T. strauchii*	30.10 ± 2.85 ^a^	-	-	-	151.40 ± 25.56 ^a^	159.1 ± 8.43 ^a^	178.50 ± 10.95 ^a^
	*T. tenuis*	13.80 ± 2.62 ^b^	-	-	-	100.4 ± 12.17 ^b^	109.90 ± 7.95 ^b^	117.80 ± 9.62 ^b^
Intestinal villi number/mm^2^	*T. strauchii*	-	-	-	-	18.40 ± 2.07 ^a^	18.90 ± 3.07	12.70 ± 1.25 ^a^
	*T. tenuis*	-	-	-	-	24.60 ± 2.17 ^b^	20.10 ± 3.14	15.00 ± 1.76 ^b^

Note: Data are presented as mean ± standard deviation (mean ± SD). For the same morphological index within the same column, values with different superscript letters indicate significant differences between species (*p* < 0.05, independent samples *t*-test).

## Data Availability

Because the project is not finalized, a link to the data has not been made public.
